# Deep learning-based spectroscopic single-molecule localization microscopy

**DOI:** 10.1117/1.JBO.29.6.066501

**Published:** 2024-05-24

**Authors:** Sunil Kumar Gaire, Ali Daneshkhah, Ethan Flowerday, Ruyi Gong, Jane Frederick, Vadim Backman

**Affiliations:** a North Carolina Agricultural and Technical State University, Department of Electrical and Computer Engineering, Greensboro, North Carolina, United States; b Northwestern University, Department of Biomedical Engineering, Evanston, Illinois, United States; c University of Tulsa, Department of Computer Science and Cyber Security, Tulsa, Oklahoma, United States

**Keywords:** deep-learning, super-resolution microscopy, label-free, spectroscopy, nanoscopy, single-molecule localization microscopy, simultaneous multicolor imaging, spectroscopic single-molecule localization microscopy

## Abstract

**Significance:**

Spectroscopic single-molecule localization microscopy (sSMLM) takes advantage of nanoscopy and spectroscopy, enabling sub-10 nm resolution as well as simultaneous multicolor imaging of multi-labeled samples. Reconstruction of raw sSMLM data using deep learning is a promising approach for visualizing the subcellular structures at the nanoscale.

**Aim:**

Develop a novel computational approach leveraging deep learning to reconstruct both label-free and fluorescence-labeled sSMLM imaging data.

**Approach:**

We developed a two-network-model based deep learning algorithm, termed DsSMLM, to reconstruct sSMLM data. The effectiveness of DsSMLM was assessed by conducting imaging experiments on diverse samples, including label-free single-stranded DNA (ssDNA) fiber, fluorescence-labeled histone markers on COS-7 and U2OS cells, and simultaneous multicolor imaging of synthetic DNA origami nanoruler.

**Results:**

For label-free imaging, a spatial resolution of 6.22 nm was achieved on ssDNA fiber; for fluorescence-labeled imaging, DsSMLM revealed the distribution of chromatin-rich and chromatin-poor regions defined by histone markers on the cell nucleus and also offered simultaneous multicolor imaging of nanoruler samples, distinguishing two dyes labeled in three emitting points with a separation distance of 40 nm. With DsSMLM, we observed enhanced spectral profiles with 8.8% higher localization detection for single-color imaging and up to 5.05% higher localization detection for simultaneous two-color imaging.

**Conclusions:**

We demonstrate the feasibility of deep learning-based reconstruction for sSMLM imaging applicable to label-free and fluorescence-labeled sSMLM imaging data. We anticipate our technique will be a valuable tool for high-quality super-resolution imaging for a deeper understanding of DNA molecules’ photophysics and will facilitate the investigation of multiple nanoscopic cellular structures and their interactions.

## 1 Introduction

Single-molecule localization microscopy (SMLM), exemplified by techniques, such as stochastic optical reconstruction microscopy (STORM),^1^ photoactivated localization microscopy (PALM),^2^^,^^3^ point accumulation for imaging in nanoscale topography (PAINT),^4^ and other variants,^5^^,^^6^ have revolutionized the field of microscopy by transcending the spatial resolution of conventional diffraction-limited microscopy. These super-resolution techniques thus enable the imaging of subcellular structures at a nanometer-level (up to ∼20  nm) resolution. Building upon wide-field imaging principles, SMLM methods exploit the ability to precisely determine the spatial coordinates of single fluorophores provided their point spread functions (PSFs) remain non-overlap and emit a sufficient number of photons.^7^^–^^9^ Recently, spectroscopic single-molecule localization microscopy (sSMLM) has emerged that utilizes additional spectral information, often overlooked in conventional SMLM imaging.^10^^,^^11^ In sSMLM, incorporating a dispersive optical component, such as a prism or grating, emission light from the sample is split into two imaging paths, both of which are captured using a single camera. The resulting image frame consists of a spatial image representing the spatial distribution of blinking events and a spectral image containing the corresponding emission spectra. These image frames undergo a computational process to determine the spatial localization position of the fluorophores and identify the corresponding distinct spectroscopic signatures (e.g., spectral peaks or centroids) associated with each blinking molecule. By correlating the spatial localization position with its associated emission spectra, it becomes possible to distinguish molecules within closer proximity compared to standard SMLM.

The commonly used blinking dyes produce a substantial number of photons during the repeated occurrence of stochastic blinking events. Regular SMLM techniques treat all these events independently due to the lack of molecular discrimination, producing a limited localization precision of around 20 nm. However, sSMLM precisely identifies repeated stochastic emissions of the same fluorophores by leveraging spectral information. Utilizing the spectral regression algorithm^10^^,^^11^ enables precise accumulation of emitted photons from the same molecule. This implementation of the spectral regression algorithm notably improves the localization precision, empowering sSMLM to achieve sub-10 nm spatial resolution.^11^^,^^12^ In addition, it enables simultaneous multicolor imaging of multi-stained samples employing dyes with very narrow spectral separation.^13^^–^^15^ Moreover, the utilization of spectral information is extended in imaging the three-dimensional multicolor super-resolution imaging,^16^ single particle tracking,^17^^–^^19^ molecule’s polarity sensing,^20^ and detecting the quantum dots dipole orientation.^21^

Despite its promising potential, sSMLM faces two major limitations. First, due to the splitting of emission light in two optical paths (∼1:3 ratio), the number of photons collected in spatial and spectral images is relatively low, impacting both spatial and spectral precision. Recent advancements, such as the introduction of a symmetrically dispersed sSMLM system and a dual-wedge prism-based system, have aimed to address these limitations.^22^^–^^24^ These advancements enhanced the spatial and spectral precision compared to regular sSMLM while reducing transmission loss. Second, simultaneous multicolor sSMLM imaging is susceptible to cross-color contamination arising from intrinsic single-molecule fluorescence spectral heterogeneity (smFSH)^25^ of the employed fluorophores. To address this challenge, deep/machine-learning techniques have recently been proposed to minimize such spectral cross-talk.^14^^,^^26^^,^^27^ Gaire et al. developed an accelerated deep-learning (DL)-based multicolor sSMLM technique that uses fewer frames than existing techniques and minimizes the spectral cross-talk.^14^ Zhang et al. developed a fully connected deep neural network^26^ and a convolution neural network (CNN)-based deep neural network^27^ to analyze the spectral profiles of each molecular emission, reducing the misidentification of spectra during spectral classification. These techniques^26^^,^^27^ primarily emphasize the spectral classification to reduce the cross-talk. Our approach, DsSMLM (deep learning sSMLM), goes beyond these methods by employing DL to improve the localization accuracy and enhance the emission spectra for better spectroscopic analysis. These advances contributed to the overall objective of achieving higher-resolution imaging and a deeper understanding of subcellular structures at the nanoscale.

Immunostaining using organic fluorescent dyes having excellent blinking properties, high photon counts, and a high signal-to-background ratio is generally preferred for labeling subcellular structures for sSMLM imaging. However, the exogenous labeling approach introduces additional width (∼17.5  nm with primary and secondary antibodies^28^) to the surface of cellular structures due to the physical dimension of the dyes. Moreover, it may potentially introduce artifacts caused by inaccurate spatial localization due to the physical dimension of the tagged fluorescent and linker molecules^29^ as well as nonspecifically binding to other components. These limitations become particularly significant when imaging macromolecular structures, such as proteins or DNA. To overcome these challenges, our lab pioneered spectroscopic intrinsic-contrast photon-localization optical nanoscopy (SICLON), specifically designed for label-free imaging of chromatin structures.^12^^,^^29^ SICLON capitalizes on the recently discovered phenomenon of stochastic fluorescence photoswitching of biopolymers, such as DNA under visible light illumination.^29^ In this approach, the nucleotide of the DNA itself serves as the source of photon emissions.^12^ However, in label-free imaging, the intrinsic contrast provided by the blinking nucleic acid is substantially lower compared to photons collected from its labeled counterpart. Therefore, the post-processing algorithm plays a crucial role in enhancing the visualization of label-free images obtained with SICLON. In addition, the distribution of such low photons to spatial and spectral imaging channels introduces additional challenges for the post-processing of the SICLON data. Overcoming these challenges is possible by leveraging advanced deep/machine learning algorithms and tailoring unique characteristics of label-free imaging. Harnessing the advantages of label-free imaging and SILCON enables researchers to explore nanoscale structures, such as chromatin, without the need for exogenous labeling. Doing so allows for the study of various biological processes, including the organization of DNA, histones, and chromatin packing domains, without the potential artifacts and limitations associated with exogenous labeling methods.

Despite significant advancements in the optical system design of sSMLM, there has been relatively little emphasis on the image processing/reconstruction aspects. A recent development in this domain is RainbowSTORM,^30^ an open-source software tool developed for regular sSMLM data analysis and image reconstruction. However, RainbowSTORM relies on conventional SMLM post-processing tools, such as ThunderSTORM,^31^ for processing the spatial image of sSMLM data, which is primarily tailored for processing data with a relatively high photon count compared to sSMLM imaging. In the case of SICLON, the intrinsic nucleotide source emits extremely low levels of photons, further challenging the reconstruction process. Therefore, there is a need for an advanced standalone post-processing procedure on these platforms capable of handling low photon conditions and providing robust performance for high-quality super-resolution imaging.

Here, we introduce a computational approach called DsSMLM, which leverages DL for robust post-processing of both label-free and fluorescence-labeled sSMLM imaging data. Our approach addresses the challenges associated with extracting accurate spatial and spectral profiles from low-photon budget images using a two-network-based DL framework. For spatial profile extraction (localization), we employ the U-Net framework,^32^ which precisely identifies the localization position of stochastically blinking events from the low-photon budgeted spatial images. For spectral profile extraction, we utilize a deep convolution neural network (DCNN) with a skip connection.^14^^,^^33^ DCNN reconstructs the low-photon budgeted spectral PSF extracted from the spectral image, which results in improving the spectral peak detection accuracy associated with each blinking single-molecule, providing better spectral analysis. These enhancements facilitate the nanoscale imaging of intricate biological structures. The preliminary results of the article were reported previously in Ref. 34. This expanded article presents additional results and their comprehensive analysis using simulated and experimental label-free and labeled sSMLM data. These findings show the effectiveness and potential of our proposed approach in advancing the capabilities of sSMLM for various imaging applications.

## 2 Reconstruction Method

**Fig. 1:**
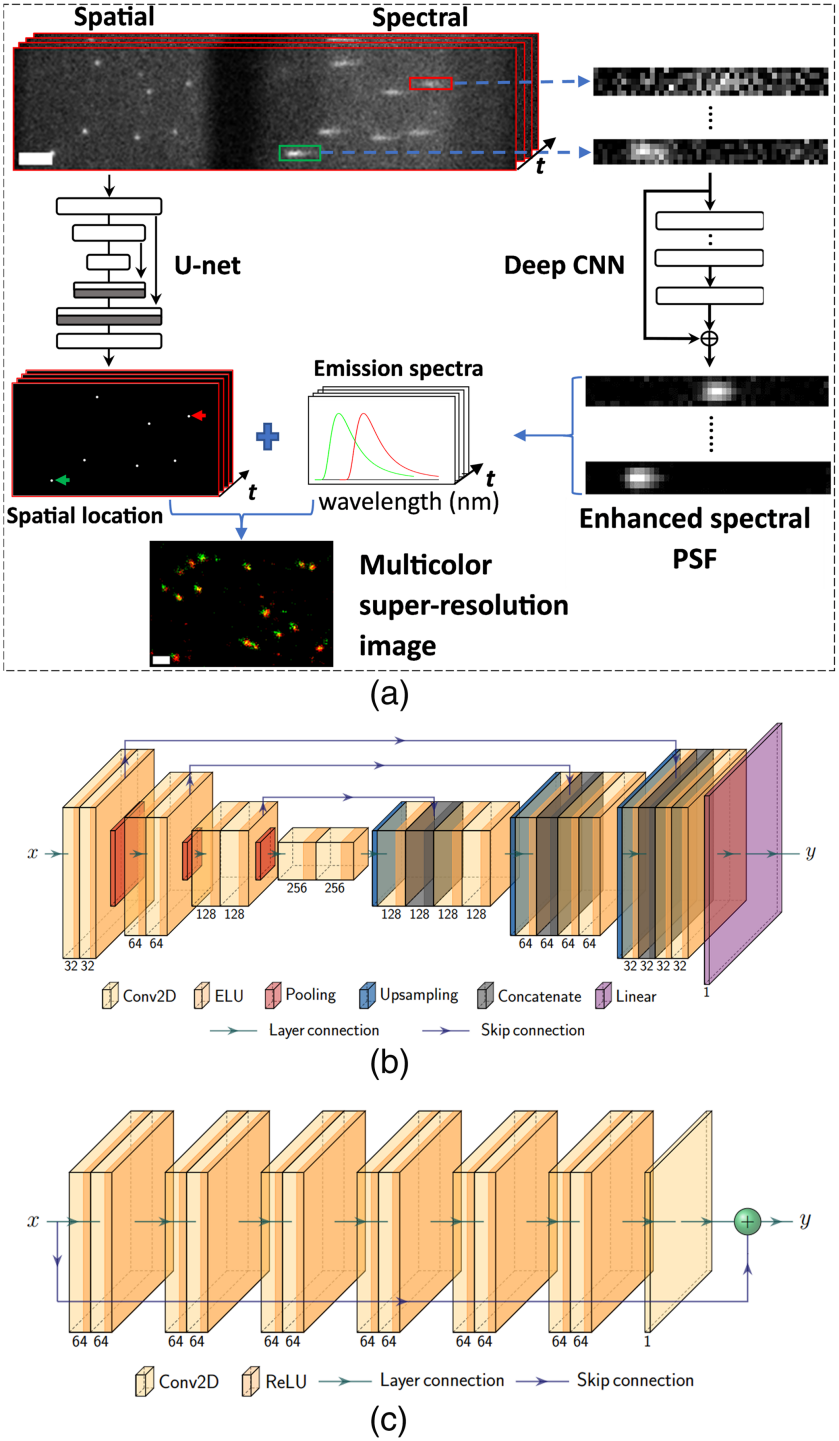
(a) Schematic of DsSMLM post-processing steps for simultaneous multicolor imaging. Scale bars=2.5  μm (frame stack) and 0.25  μm (multicolor image). (b) and (c) The detailed block of U-Net architecture for spatial PSFs localizations and DCNN architecture for enhancement of spectral PSFs.

In sSMLM, an experimentally recorded diffraction-limited frame consists of spatial and spectral images, where the spatial image provides the spatial location of blinking single-molecules and the spectral image provides the corresponding emission spectra. DsSMLM utilizes the U-Net framework to localize the blinking PSFs precisely on the low-photon budget spatial images. The localization coordinates are obtained by extracting the local maxima position of predicted positions from the U-Net predicted images.^35^^,^^36^ In addition, DsSMLM uses DCNN to reconstruct the low-photon budgeted spectral PSFs of spectral images. The region of interest (ROIs) of spectral PSFs for reconstruction are cropped using the estimated localization coordinates as a reference point, along with spectral calibration and spectral dispersion information from the imaging system. The DCNN reconstructed spectral PSFs produce an accurate spectral profile (e.g., spectral peak value) for the spectral analysis [Figs. 8(f) and 8(g)]. The overall post-processing steps, as shown in Fig. 1(a), involve processing both the spatial (using U-Net) and spectral (using DCNN) images from several thousands of frames. This leads to a list of localizations along with their corresponding wavelengths information $(F_{i},x_{i},y_{i},\lambda_{i}{)}_{i=1...n}$ where $F_{i}\in [1,N]$ represents the index of diffraction-limited frames from which localization $\left(x_{i},y_{i}\right)$ originates; $\lambda_{i}$ denotes the distinct emission spectra at that location; N represents the total frame number; and n is the total number of localizations. Further post-processing steps include filtering out localizations originating from fluorescent impurities, such as background noise, auto-fluorescence, or nonspecific binding.^37^ More specifically, the localizations having wavelength values out of the spectral window of the labeled dye or DNA molecules are filtered. The lateral drift of blinking positions introduced during imaging is corrected using the cross-correlation-based drift correction method,^31^^,^^38^ and the spectral regression algorithm^10^ is applied to improve the spatial resolution. Using DsSMLM’s unique and precise identification capability of the repeated stochastic emissions of the same fluorophores with the help of spectral information, the spectral regression algorithm accumulates all the emitted photons from the same molecule, providing improved localization precision. The resulting post-processed localizations are then used to render the final super-resolution image.

In labeled multicolor super-resolution imaging, the retrieved spectral information is utilized to separate the post-processed localizations list into multiple imaging channels according to the pre-defined spectral window of labeled dyes. Finally, the composite multicolor super-resolution image is generated by combining the extracted images from all imaging channels.

### 2.1 DsSMLM Training and Test Data

Both DL models (U-Net and DCNN) used in DsSMLM were trained on simulated data. Specifically, we simulated the sSMLM experimental image frames and corresponding ground truth image frames. The simulation process involved generating the spatial diffraction-limited image frames with sparse and randomly distributed two-dimensional Gaussian PSFs in each frame. A low emitter density ($0.1\;\mathrm{molecules}/\mu m^{2}$ per frame) was used to avoid excessive overlapping of PSFs. Then, the spectral image frames were generated by convolution of each PSF with the emission spectra of the specific fluorescent molecules, such as Alexa Fluor 647, at a certain spectral dispersion. Shot noise and background noise were added to generate a realistic noisy image frame. The simulation parameters were chosen to closely match the characteristics of the experimental data.^39^ Following this procedure, we generate a simulated sSMLM image stack having 1.3K frames. From this simulated frame stack, two sets of training data were generated for training the U-Net and DCNN separately.

**Fig. 2:**
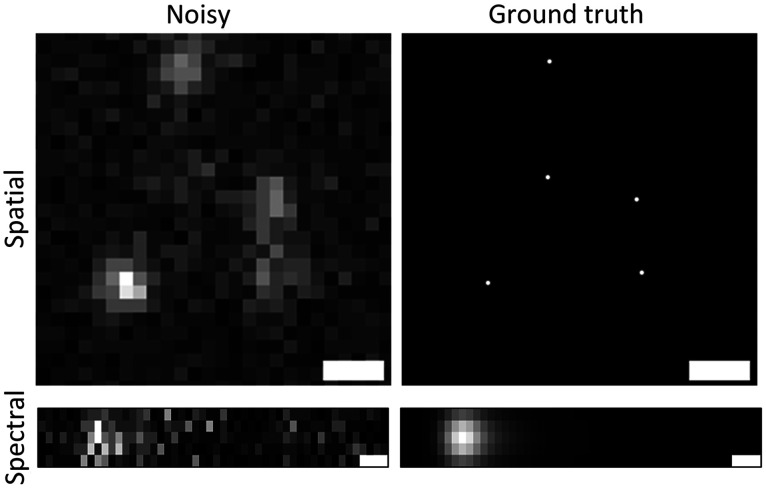
Sample simulated training data pair (input and ground truth) for localization of PSFs in the spatial image and enhancing the emission spectra from the spectral image. Scale bar=0.5  μm.

For the U-Net training, the spatial image stack with a size of 256×256  pixels was cropped from the simulated data. Instead of directly using the full-size spatial images from the stack, we generated overlapping patches of size 208×208  pixels. Overall, 15K image patches, each having at least three emitters, were used for training. We also extracted the respective (x,y) emitter positions. The ground truth of the spatial frame was generated by projecting the positions to the upsampled high-resolution image grid. Alternatively, this training data can also be generated by using existing SMLM tools, such as ThunderSTORM,^31^ as used in Deep-STORM.^40^ A sample training data pair for U-Net (spatial) is shown in Fig. 2.

For the DCNN training, spectral PSFs ROI were cropped from the simulated spectral image stack, as shown in Fig. 1(a). Specifically, ROI containing the spectral PSFs from simulated noise-free (ground-truth) and noisy spectral images were cropped to generate the training data pair. A sample training data pair for DCNN training (spectral) is shown in Fig. 2. The label-free and labeled data have slightly different spectral dispersion, so the image size of the training data was slightly different. We used ROIs of pixel size 9×95 for the label-free and a pixel size of 5×50 for training all the labeled images. Overall, 143K image pairs were used to train this model.

For testing, simulated as well as actual experimental data were used. The sample preparation and imaging of experimental data are explained in Sec. 4. The camera pixel sizes of our experimental label-free and labeled data are different, so separate training data were generated for each of them.

### 2.2 Network Architectures, Training, and Testing Strategies

DL has been a versatile and powerful tool for various imaging applications,^41^ including image reconstruction and analysis in optical microscopy.^35^^,^^42^^–^^46^ Convolution neural networks (CNNs) have recently been used to detect and identify emitter coordinates,^36^^,^^40^^,^^47^^,^^48^ color extraction,^49^^,^^50^ background estimation,^51^ reconstruction of high-density images,^14^^,^^52^ and to design optimized PSFs.^47^^,^^50^ Building upon this trend, DsSMLM incorporates two-network-model-based post-processing approaches for the reconstruction of sSMLM data. Accurately detecting and estimating the brightest pixel position of each blinking PSF by ignoring the background noise on the spatial image frame resembles the detection and localization task. The U-Net framework is a well-established model for such tasks, so we use a U-Net to detect and localize the spatial PSFs. The diffraction-limited image is input to the U-Net, which outputs an image with predicted localization positions. The U-Net architecture consists of an encoder and a decoder with skip connections, enabling the mapping of features between them. In our implementation [Fig. 1(b)], the encoder contains the repeated application of two convolution layers with a kernel size of 3, followed by a max-pooling layer having a kernel size of 2. Thus, each downsampling step at the encoder results in the feature maps with half size and double number. The number of filters is shown at the bottom of each convolution layer in Fig. 1(b). Every step in the decoder consists of upsampling convolution layers with a kernel size of 2, resulting in feature maps of doubled size and halved number. Then, at each step, the output of each downsampling is concatenated with the result of the upsampling at corresponding spatial scales. The concatenated output is then followed by two convolution layers of kernel size 3. The last layer of the decoder network, with a kernel size of 1, generates the pixelwise predicted image. The bottleneck layer resides between the encoder and decoder and consists of two convolution layers with a kernel size of 3. We used exponential linear unit (ELU)^53^ as an activation function for all convolution layers except for the last one, which uses a linear activation function. This model is trained by minimizing the combined mean squared error (MSE) and mean absolute error loss function ($l_{\mathrm{loc}}$).^40^

$$l_{\mathrm{loc}}=\frac{1}{n}\overset{n}{\underset{i=1}{\sum }}{\left\|x_{i}⊛g-y_{i}⊛g\right\|}^{2}+{\left\|y_{i}\right\|}_{1},$$ (1)

where n is the number of training image pairs; ⊛ is the convolution; $y_{i}=f(x_{i};\Theta )$ is the i’th network prediction for input $x_{i}$, where Θ is the DL network parameters to be learned during training; and g is the Gaussian kernel.

The spectral PSFs having low photon count [as shown in Fig. 1(a)] need to be reconstructed for accurate spectral signature extraction. Thus, the spectral PSF enhancement task can be formulated as an image restoration task. DCNN with skip connection is well known for such restoration applications.^14^^,^^54^ So, DsSMLM employed DCNN to enhance the emission spectra. Our DCNN implementation [Fig. 1(c)] consists of seven weighted convolution layers with a skip connection. The input and output are the cropped spectral PSFs. For each layer except the last one, 64 filters with a kernel size of 3 are used. The first layer operates on the input image, and the successive layers learn the feature maps from the input image. The last layer, consisting of a single filter with a kernel size of 3, is used for image reconstruction. For all layers except the last one, the rectified linear units (ReLU) are used as an activation function. The MSE is used as an enhancement loss function ($l_{\mathrm{enh}}$) for training this model:

$$l_{\mathrm{enh}}=\frac{1}{n}\overset{n}{\underset{i=1}{\sum }}{\left\|x_{i}-y_{i}\right\|}^{2},$$ (2)

where n is the number of training image pairs, and $y_{i}$ is the network prediction for the i’th input $x_{i}$.

Both networks were trained separately using the Adam optimizer in Keras with the TensorFlow platform and implemented in the Python framework. During training, the training data were split into a 90:10 ratio for training and validation purposes. The network training and testing were performed on a Dell workstation with NVIDIA’s RTX A4000 graphical processing units. U-Net was trained for 200 epochs with a network training time of over 3 h. Similarly, DCNN was trained for 30 epochs with a training time of about 1 h.

## 3 Optical System Setup and Calibration

**Fig. 3:**
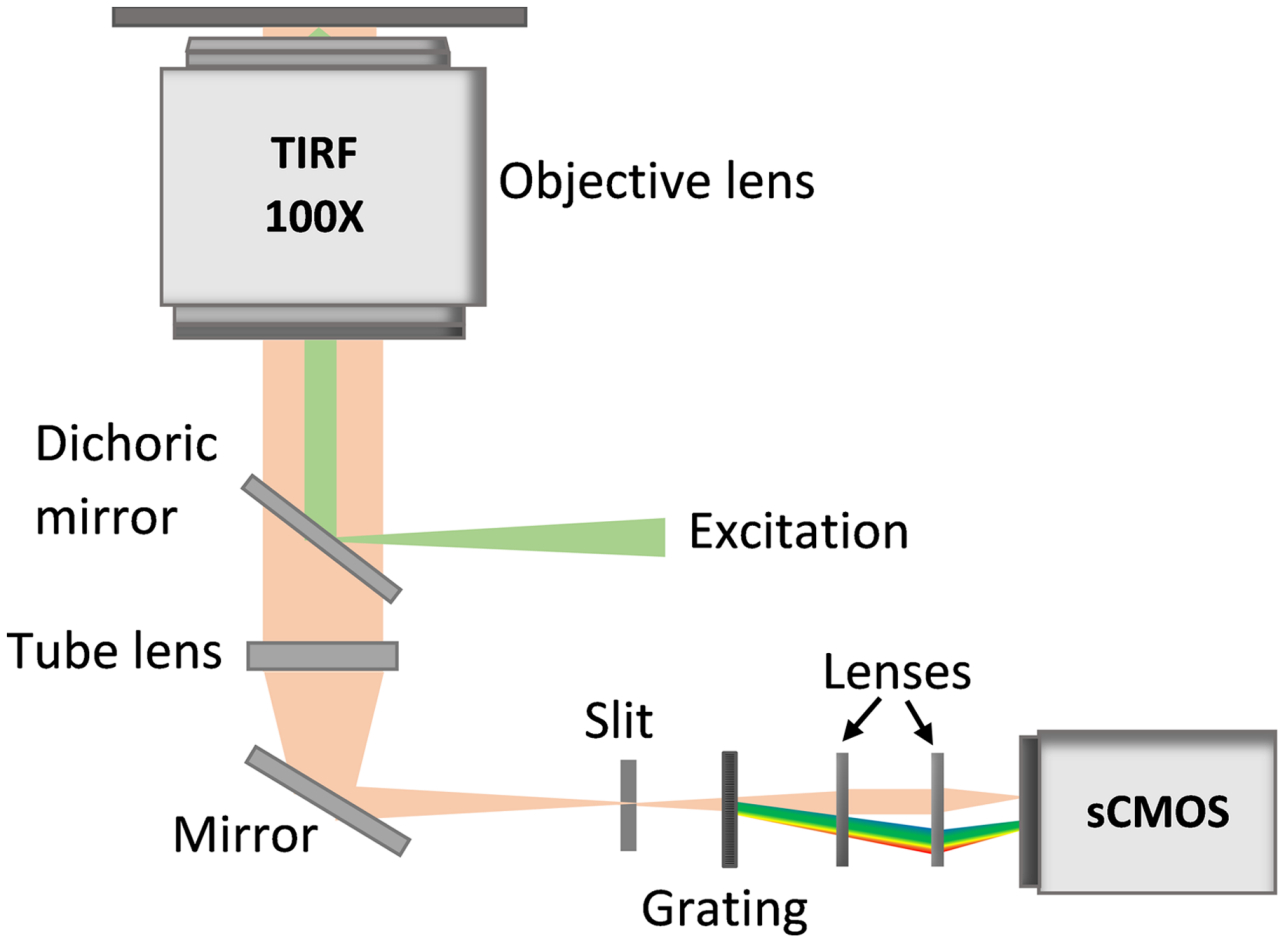
A simplified schematic of the sSMLM imaging setup.

The sSMLM experimental system utilized in this study has been previously described in detail in Refs. 10, 12, and 55. In summary, the system consists of five continuous-wave lasers employed for excitation (405, 488, 532, 552, and 637 nm, (110, 165, 165, 22, 154 mW, OBIS Laser Box, Coherent, California)). The laser beam was guided into an inverted microscope (Ti-E, Nikon, Tokyo, Japan) and focused by a lens into the back focal plane of a total internal reflection objective (Nikon 100×, numerical aperture = 1.49). A transmission diffraction grating with 100 lines per mm (Star analyser 100, Paton Hawksley Education Ltd., Bristol, United Kingdom) was used to split each frame into zeroth- and first-order channels (1:3 ratio). The zeroth-order channel provides the unmodified spatial images, and the first-order channel provides the spectrally dispersed first-order spectral images. Both the spatial and spectral images were then simultaneously captured by the same scientific complementary metal-oxide-semiconductor (sCMOS) camera (Prime 95B, Photometrics, Teledyne, Arizona). Low illumination power was utilized at the back focal plane, and the exposure time of 20 to 30 ms was used for image acquisition. A simplified schematic of the optical setup is shown in Fig. 3.

**Fig. 4:**
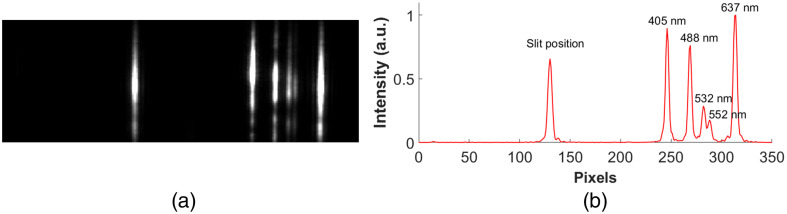
Calibration of sSMLM system. (a) Reference calibration image. (b) Line profile of (a) showing calibrated spectra. The first peak corresponds to the slit position, and the multiple peaks on the right represent the known emission spectra of the light source.

A spectral calibration procedure is required to obtain the emission spectra of single molecules from the recorded spectral images and is specific to individual sSMLM systems. Using the calibration light source (multiple laser lines), reference images were captured, which included a straight line confined by a narrow slit and multiple spectral lines [Fig. 4(a)]. The straight line in the spatial image corresponds to the slit/emitter position, while the multiple spectral lines in the spectral images represent the known emission maxima (405, 488, 532, 552, and 637 nm) related to the calibration light source [Fig. 4(b)]. The peak pixel positions are then linearly fitted to the known wavelength.

## 4 Sample Preparation and Image Acquisition

### 4.1 Label-Free SICLON Imaging

The label-free imaging data of isolated salmon sperm single-stranded DNA (ssDNA, Sigma-Aldrich, St. Louis, Missouri) in its unmodified form were taken from Ref. 12. In summary, the imaging experiment includes the suspension of ssDNA in nuclease-free water (Thermo Fisher Scientific, Waltham, Massachusetts) and then spin-coating on a glass coverslip, producing linearly deposited ssDNA fiber.^12^^,^^29^ Then, unlabeled ssDNA fiber was imaged using the 532 nm laser excitation. A total of 5K image frames using the sSMLM system were recorded using an electron multiplying charge-coupled device camera (ProEM512B Excelon, Princeton Instruments, New Jersey).

### 4.2 Cell Culture, Labeling, and Image Acquisition for Fluorescence-Stained sSMLM Imaging

For the labeled sSMLM imaging experiments, immunostaining was performed on COS-7 (#CRL-1651) and U2OS (#HTB-96) cells (American Type Culture Collection, Virginia). COS-7 cells were cultured with Dulbecco’s modified eagle’s medium (#11965092, Thermo Fisher Scientific, Waltham, Massachusetts), and U2OS cells were cultured in McCoy’s 5A Modified Medium (#16600082, Thermo Fisher Scientific, Waltham, Massachusetts). Both culture media were supplemented with 10% fetal bovine serum (#26140095, Thermo Fisher Scientific, Waltham, Massachusetts) and 100  μg/mL penicillin-streptomycin (#15140122, Thermo Fisher Scientific, Waltham, Massachusetts). Cells were plated on 35 mm glass bottom dishes (#D35-14-1-N, Cellvis, Mountain View, California) at 25K to 50K seeding density and allowed to grow for ∼48  h at physiological conditions (37°C in 5% ${\mathrm{CO}}_{2}$). Then, the cells were fixed in 3% paraformaldehyde (#50-980-487, Electron Microscopy Sciences, Hatfield, Pennsylvania) and 0.1% glutaraldehyde (GA, #G5882, Sigma-Aldrich, St. Louis, Missouri) in phosphate buffer saline (PBS, #10010023, Thermo Fisher Scientific, Waltham, Massachusetts) for 10 min. The cells were then washed with PBS once, quenched with freshly prepared 0.1% sodium borohydride (${\mathrm{NaBH}}_{4}$, #S678-10, Fisher Scientific, Waltham, Massachusetts) in PBS for 7 min, and rinsed with PBS three times. The fixed cells were then permeabilized with a blocking buffer containing 3% bovine serum albumin (BSA, #A3294, Sigma-Aldrich, St. Louis, Missouri), 0.2% Triton X-100 (#28314, Thermo Fisher Scientific, Waltham, Massachusetts) and PBS for 20 min at room temperature and then incubated with primary antibodies for H3K9me3 (rabbit anti-histone H3K9, $2.5\;\mu g\;{\mathrm{mL}}^{-1}$, #ab176916, Abcam, Cambridge, United Kingdom) or H3K4me3 (mouse anti-histone H3K4, $2.5\;\mu g\;{\mathrm{mL}}^{-1}$, #ab12209, Abcam, Cambridge, United Kingdom) in blocking buffer for 1 to 2 h at room temperature or overnight at 4°C and rinsed with a washing buffer (0.2% BSA, 0.1% Triton X-100 in PBS) for three times. The cells were further incubated with corresponding goat secondary antibody conjugated with Alexa Fluor 647 (AF647, $2.5\;\mu g\;{\mathrm{mL}}^{-1}$, Thermo Fisher Scientific, Waltham, Massachusetts) and CF680 ($2.5\;\mu g\;{\mathrm{mL}}^{-1}$, Biotium, Fremont, California) for 40 min, washed thoroughly with PBS three times at 25°C, and stored at 4°C.

Before imaging, an imaging buffer containing 50 mM Tris (pH = 8.0, #T3038, Sigma-Aldrich, St. Louis, Missouri), 10 mM NaCl (#721016, Thermo Fisher Scientific, Waltham, Massachusetts), $0.5\;\mathrm{mg}\;{\mathrm{mL}}^{-1}$ glucose oxidase (#G2133, Sigma-Aldrich, St. Louis, Missouri), catalase (#C100, Sigma-Aldrich, St. Louis, Missouri), 10% (w/v) D-glucose (#15023021, Thermo Fisher Scientific, Waltham, Massachusetts), and either 2-mercaptoethanol (#63689, Sigma-Aldrich, St. Louis, Missouri) or 100 mM cysteamine (#C121509, Sigma-Aldrich, St. Louis, Missouri) were added to each plate. We recorded 10K image frames for each sSMLM image acquisition with an exposure time of 20 to 30 ms.

### 4.3 Image Acquisition for Simultaneous Multicolor sSMLM Imaging

For the simultaneous multicolor imaging experiment, we used a commercially available DNA origami nanoruler (GATTA-PAINT 40RG, GATTAquant, Munich, Germany) sample.^56^ This nanoruler sample has a mark-to-mark distance of 40 nm and is labeled with two dyes: ATTO 542 and ATTO 655. The imaging was performed according to the manufacturer’s recommendations. During imaging, both dyes in the sample were simultaneously excited using two laser sources at 532 and 637 nm. We then recorded 5K frames with an exposure time of 30 ms.

## 5 Reconstruction Results

### 5.1 DsSMLM Performance on Simulated Data

**Fig. 5:**
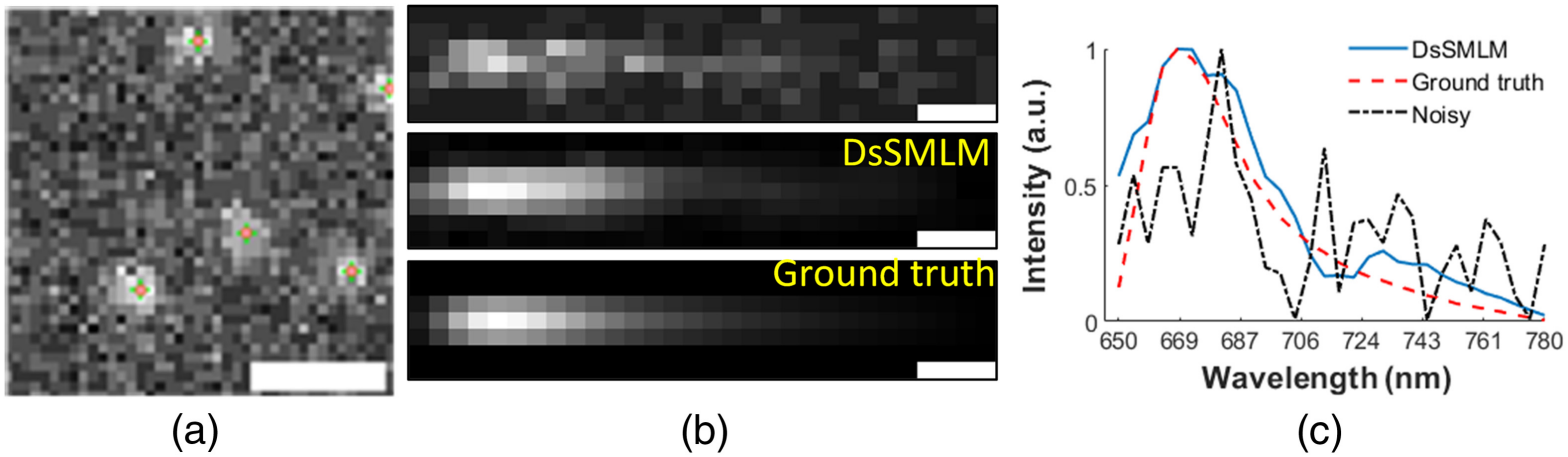
Performance of DsSMLM on simulated data. (a) Representative simulated spatial frame with ground truth coordinates (green plus) and the DsSMLM predicted coordinates (orange circle). Scale bar=1  μm. (b) Representative DsSMLM reconstructed spectral PSF compared with simulated and ground truth spectral PSF of AF647 dye. Scale bars=0.5  μm. (c) Spectral plots of images in (b). Noisy is the spectrum of simulated PSF. The emission spectrum from DsSMLM is smooth and very close to the ground-truth image with the matching peak.

We evaluated the performance of both DL models used in DsSMLM separately using the new simulated data that were not used in the training process. The simulated data were generated following the same procedures as explained in Sec. 2.1. Figure 5(a) shows a representative simulated spatial image frame with the ground truth coordinates (green plus) and the DsSMLM predicted coordinates (orange circle). We used 100 similar frames of size 64×64  pixels and performed the quantitative evaluation of the DsSMLM localization results by using the commonly used evaluation matrices Jaccard index (JI) and lateral root mean squared error (RMSE).^36^^,^^57^ JI is defined as

$$\mathrm{JI}=\frac{\mathrm{TP}}{\mathrm{FN}+\mathrm{FP}+\mathrm{TP}},$$ (3)

where TP are the true positives (number of localizations matched to the ground truth coordinates), FN are the false negatives (number of undetected ground truth coordinates), and FP are the false positives (number of spurious localization coordinates). Similarly, lateral RMSE is defined as

$$\text{Lateral }\mathrm{RMSE}={\left[\frac{1}{\mathrm{TP}}\overset{\mathrm{TP}}{\underset{i=1}{\sum }}{\left(x_{i}-x_{i}^{\mathrm{GT}}\right)}^{2}+{\left(y_{i}-y_{i}^{\mathrm{GT}}\right)}^{2}\right]}^{\frac{1}{2}},$$ (4)

where $\left(x_{i},y_{i}\right)$ represents the predicted localization coordinates, and $\left(x_{i}^{\mathrm{GT}},y_{i}^{\mathrm{GT}}\right)$ represents the ground truth localization coordinates. JI measures the fractions of correctly detected emitters, quantifying the detection accuracy, and the score value ranges from 0 to 1 with a theoretical optimum value of 1. The localization error quantified by the lateral RMSE gives the root mean square distance between the ground truth and detected localization positions. We measured the emitter detection considering the lateral threshold radius of 100 nm. The JI and lateral RMSE from our localization results were 0.734 and 28.334 nm, respectively. The high JI and low lateral RMSE values show our method performed well in the simulation environment. Figure 5(b) shows the representative image of DsSMLM reconstructed spectral PSF along with the ground truth and simulated spectral PSF images of AF647 dye. For the quantitative evaluation of spectral PSF reconstruction, we used the structural similarity index (SSIM), which evaluates the reconstruction capability to capture the structural information compared with the ground truth image. The SSIM index has a scale between 0 and 1, with 1 being a perfect match with the ground-truth image. The higher SSIM value indicates a better match of structural information. The SSIM values were 0.5440 and 0.8187, respectively, for simulated and DsSMLM reconstructed images. We then extracted the spectral signatures from all three images. The plots in Fig. 5(c) were obtained by taking the mean pixel value of each spectral PSF of Fig. 5(b) in the y direction and normalizing them to create a one-dimensional outline of each spectrum. Corresponding wavelength values are obtained using the calibration data, using spatial PSF localization position as the reference point. DsSMLM reconstructed spectral profile is close to the ground truth profile. We then search the peak value of these profiles using the peak finding algorithm, and the corresponding wavelength value is the spectral peak. DsSMLM and ground truth images have the same spectral peak of 672.40 nm, while the noisy simulated image peak value is 685.84 nm. Such accurate spectral peak prediction using DsSMLM improves the reconstruction quality of spectroscopic super-resolution imaging data.

**Fig. 6:**
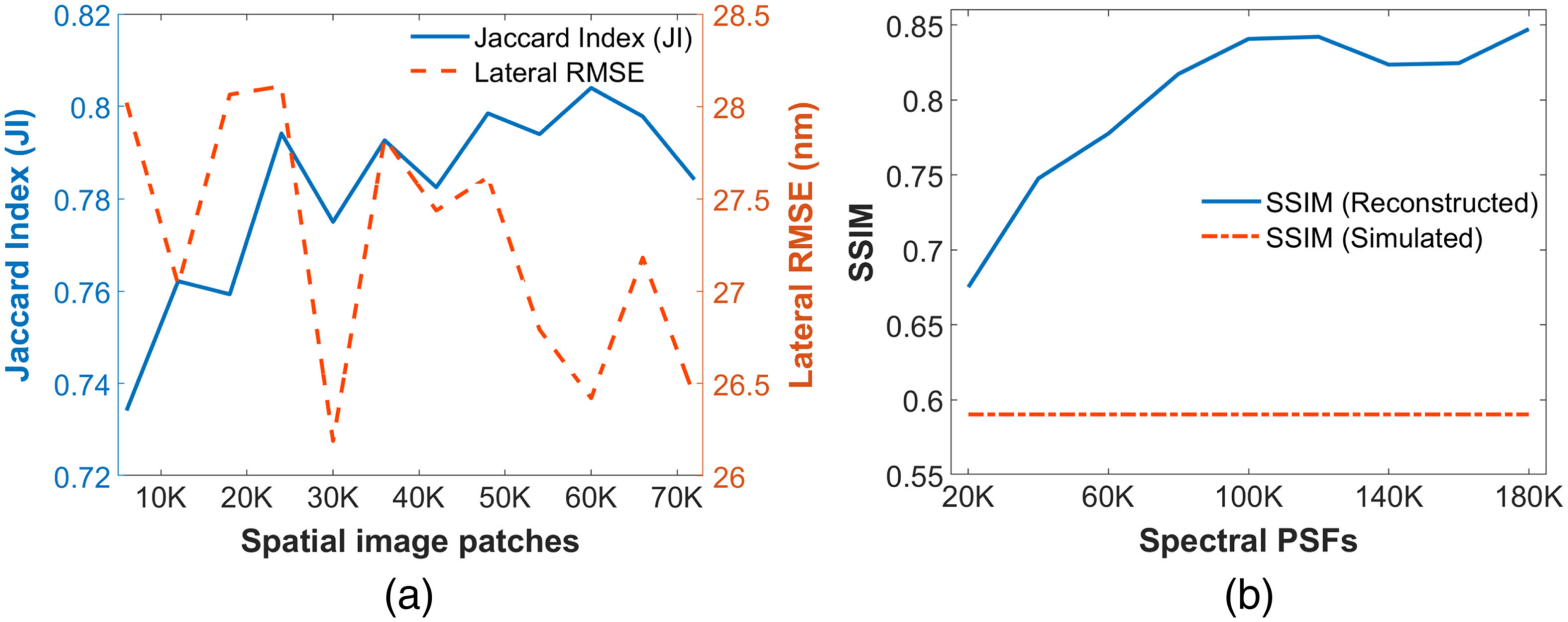
Evaluation of DsSMLM performance on different numbers of training data used. (a) Localization performance (JI and lateral RMSE) with an increasing number of spatial image patches. (b) Spectral enhancement performance (SSIM) with an increasing number of spectral PSF data.

We further evaluate the performance of both DL models based on the amount of training data used. Figure 6(a) quantifies the blinking emitter detection and localization accuracy with an increasing number of training datasets. With >12  K training data, the JI is higher than 0.75, which signifies that the model is detecting emitters very well with fewer false positives. There is a slight variation in the value of lateral RMSE over different training data, but the overall value remained less than 28.1 nm, signifying very less localization error. Similarly, Fig. 6(b) quantifies the spectral PSF reconstruction performance measured with the SSIM index for the increasing training data size. As expected, the reconstruction performance increased significantly with a higher amount of training data. After 80K training samples, the SSIM value for reconstruction is higher than 0.80, which implies higher structural similarities of reconstructed spectral PSFs with ground truth PSFs. This enhancement of the spectral profile gives accurate spectral signatures of each blinking emitter, providing an accurate spectral analysis. The SSIM values for the raw simulated PSF data were consistent with ∼0.59 over the whole range. These performances were evaluated on five different simulated sSMLM test images with 100 frames, and mean measurement values from these five samples were reported.

### 5.2 DsSMLM for Label-Free sSMLM Imaging

**Fig. 7:**
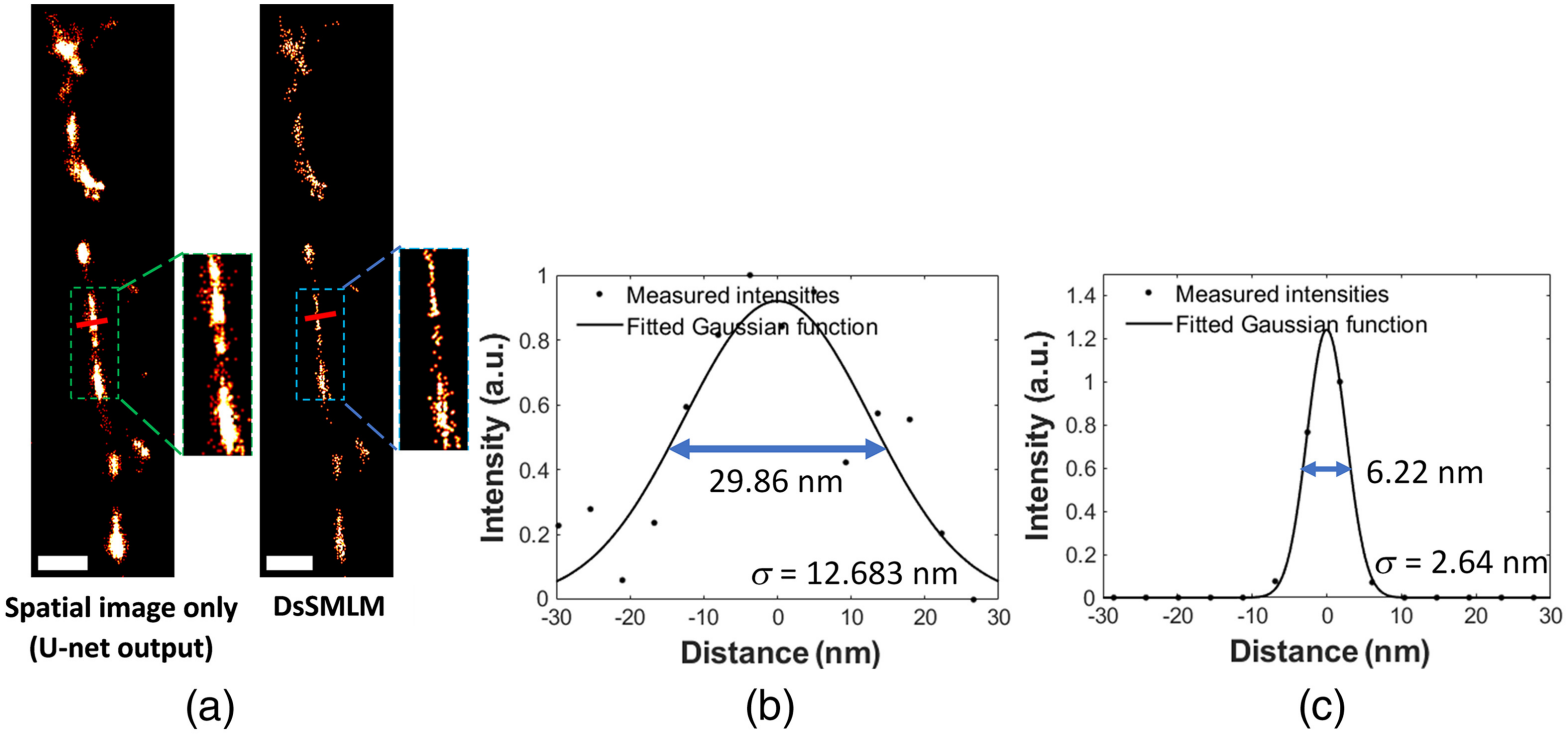
(a) The DsSMLM reconstructed label-free intrinsic contrast ssDNA fiber image (right) compared with the image obtained at the U-Net output (left). The image on the left was obtained by rendering the localization from the U-Net output, analyzing the spatial images only. Pixel size = 4.3 nm, scale bar = 300 nm. (b) and (c) Intensity profile and FWHM of the red line segments shown in (a) for the spatial image only (left) and DsSMLM (right), respectively. Black dots are measured intensities, and black curves are fitted Gaussian functions with standard deviation σ and FWHM (blue double arrow) as indicated. Due to the low photon budget in the spatial images, the precision (and resolution) is relatively low in (b). Such low precision was improved by analyzing both spatial and spectral frames in DsSMLM, achieving a high resolution in (c).

The reconstruction result of the label-free ssDNA using DsSMLM, shown in Fig. 7(a), reveals the characteristic linear features inherent in DNA fibers. These features arise from the nucleic acids present in the sample. To access the resolution of the reconstructed image, we analyzed the full width at half maximum (FWHM) based on the intensity profile along the red line segment of the filament, as indicated in Fig. 7(a). FWHM evaluations were conducted on the reconstruction results: (1) using only the spatial image frames processed by the U-Net [Fig. 7(a), left] and (2) both spatial and spectral image frame data processed by DsSMLM [Fig. 7(a), right]. Reconstruction utilizing only the spatial image frame results in a resolution of 29.86 nm [Fig. 7(b)], while processing both spatial and spectral images using DsSMLM results in a resolution of 6.22 nm [Fig. 7(c)]. The FWHM was calculated using $2\sqrt{2\;\ln\;2}\sigma \approx 2.355\sigma$, where σ is the standard deviation (s.d.) of the measured intensities (black dots). As shown in Fig. 7(b), if only the spatial image is analyzed, the precision is relatively low due to the low photon budget in the spatial image. This challenge is addressed through the integration of spectral data. Using spectral information, DsSMLM precisely identifies the repeated stochastic emission of the same molecules from different image frames and accumulates them using the spectral regression algorithm, contributing to improved precision and resolution. Thus, about a 4.8-fold improvement in resolution was achieved using DsSMLM compared to the analysis of spatial information only. The outstanding resolution of 6.22 nm achieved by DsSMLM is an improved performance of ∼2.28  nm compared to our previous findings of 8.5 nm reported in Ref. 12. It brings us closer to the resolution of 4.1 nm observed with atomic force microscopy.^12^ Therefore, when combined with novel label-free imaging, DsSMLM enables the characterization of nanoscale structures at the sub-10 nm region, surpassing the capabilities of conventional SMLM.

### 5.3 DsSMLM for Fluorescence-Stained sSMLM Imaging

**Fig. 8:**
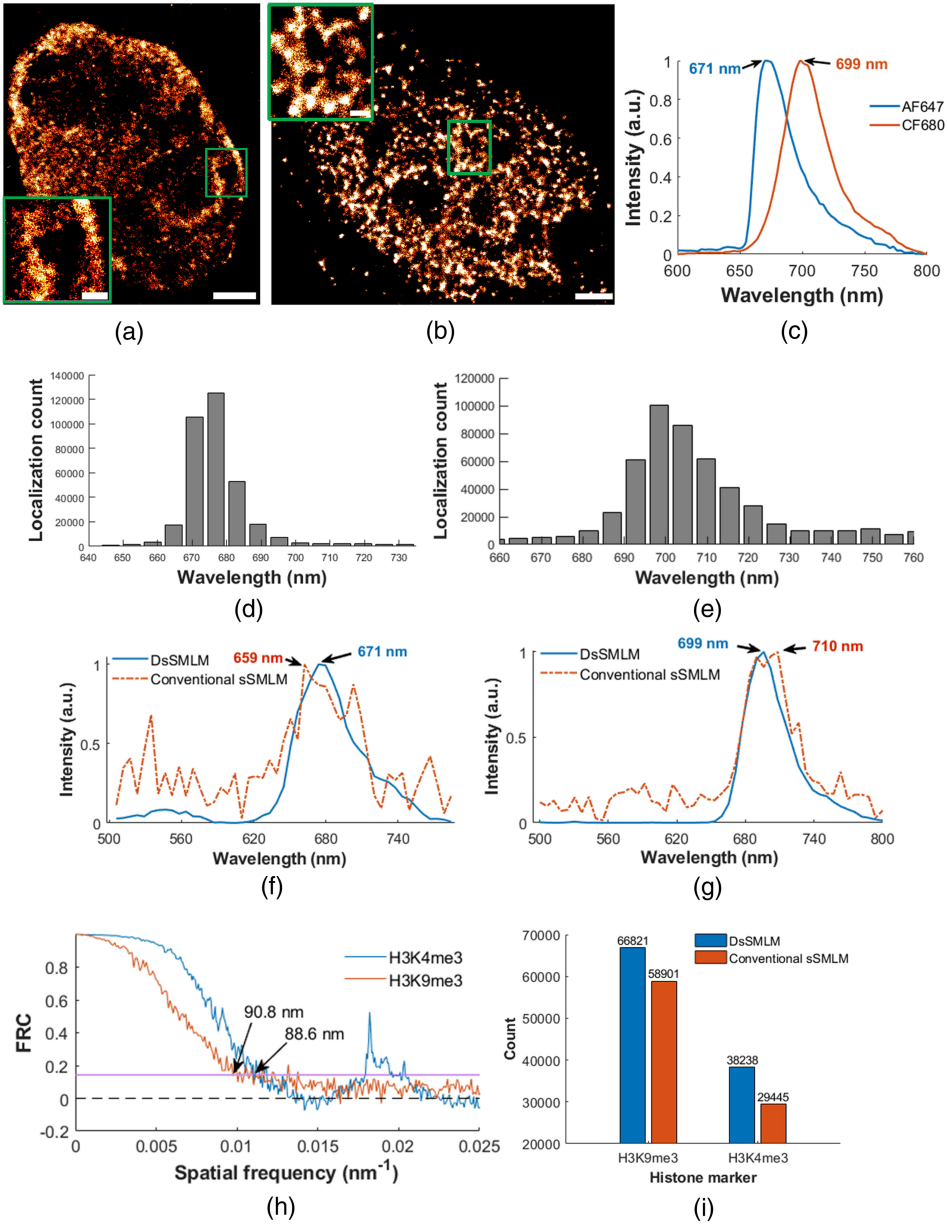
(a) and (b) DsSMLM reconstruction of H3K9me3 and H3K4me3 histone marker in COS-7 and U2OS cells labeled separately with AF647 and CF680 dyes, respectively. Scale bar=2  μm and 500 nm for inset. (c) The normalized emission spectra with their spectral peak values of labeled dyes. (d) and (e) Localization counts with their spectral peak positions of figures (a) and (b). (f) and (g) Sample spectral plot using the DsSMLM and conventional sSMLM. (h) FRC resolution of images shown in panels (a) and (b). The pink line denotes the fixed threshold value of 1/7. (i) Accurately predicted spectral peaks count within the spectral window of 20 nm using the DsSMLM and conventional sSMLM. The mean count values were obtained from 100K randomly selected spectra in 10 observations.

We evaluated the performance of our DsSMLM algorithm for the reconstruction of higher-order chromatin structures labeled with fluorescence dyes. Figure 8(a) shows the super-resolution reconstructed image of the transcriptionally repressive histone marker, H3K9me3 (constitutive heterochromatin), labeled with AF647 dye in the whole nucleus of a COS-7 cell. The field-of-view (FOV) of the image is 11.80  μm×13.18  μm, and ∼380K localizations were used to reconstruct the super-resolution image. The result reveals that the distribution of the H3K9 trimethylation marks is primarily enriched at the periphery of the nucleus, nucleolus, and nucleoplasm. Similarly, Fig. 8(b) displays the super-resolution image of the transcriptionally active histone marker, H3K4me3 (euchromatin), in U2OS cells labeled with CF680 dye. The FOV of the image is 18.59  μm×15.95  μm, and ∼350K localizations were utilized to reconstruct the super-resolution image. The active histone marker appears to be localized and distributed on the inner side of the nucleus. The green boxes in Figs. 8(a) and 8(b) highlight the segregated accumulation within the H3K9 and H3K4 protein-rich regions. Such a nanoscale visualization of chromatin-rich and chromatin-poor regions offered by DsSMLM will be a valuable tool for understanding chromatin biology.

The spectral peak distribution of each blinking event associated with Figs. 8(a) and 8(b) are shown in Figs. 8(d) and 8(e). The AF647 labeled sample [Fig. 8(d)] has slightly better precision than the CF680 labeled sample [Fig. 8(e)]. This betterment is attributed to the AF647 dye’s brighter and more photostable nature, enabling more accurate spectral peak detection even in low-photon conditions compared to CF680. Such measurement of spectral peak distribution of Figs. 8(d) and 8(e) allows us to filter the localizations originating from the background noise or auto-fluorescence, confirming localization from the labeled histone markers only. In this case, we use a filter size of 60 nm (640 to 700 nm for AF647 and 670 to 730 nm for CF680 dyes) to perform such filtering. Figures 8(f) and 8(g) show normalized DsSMLM reconstructed spectra samples with their associated spectral peaks for AF647 and CF680 labeled samples compared with conventional sSMLM reconstruction. DsSMLM reconstructed spectra and their measured peaks are very close to the ground-truth spectra of Fig. 8(c) for both cases. The ground truth emission peak value of the AF647 dye is ∼671  nm and that of the CF680 dye is ∼699  nm. Notably, DsSMLM is able to correct the deviated spectral peak of about 12 nm. This accurate spectral prediction improves confidence in reconstruction, minimizing artifacts in labeled super-resolution imaging. We also measured the Fourier ring correlation (FRC)^58^ resolution of the DsSMLM reconstructed images of histone markers [Fig. 8(h)]. The FRC resolution was 90.8±5.36  nm and 88.6±3.07  nm for the images shown in Figs. 8(a) and 8(b), respectively.

Further, we evaluated the robustness and accuracy of DsSMLM in identifying the precise spectral peaks of labeled dyes in H3K9me3 and H3K4me3 histone markers. To do so, we measured the spectral peak of randomly selected 100K emission events from each histone marker and counted the localization whose spectral peak lies within the spectral window of 20 nm, as shown in Fig. 8(i). In the case of H3K9me3, DsSMLM identified an average of 66821 localizations with a s.d. of 4923, while the conventional method distinguished an average of 58901 localizations with a s.d. of 2218. This indicates that DsSMLM outperformed the conventional method by identifying ∼7.9% (7.9K) more localizations. Similarly, for H3K4me3, DsSMLM identified an average of 38238 localizations with a s.d. of 139, while the conventional method identified an average of 29445 localizations with a s.d. of 3102. Thus, DsSMLM achieved ∼8.8% (8.7K) higher localizations compared to the conventional method. These average and s.d. values were calculated for 10 random observations. The higher localization counts coupled with accurate spectral peak detection using DsSMLM ultimately improve the quality of the final super-resolution image.

### 5.4 DsSMLM for Simultaneous Multicolor sSMLM Imaging

**Fig. 9:**
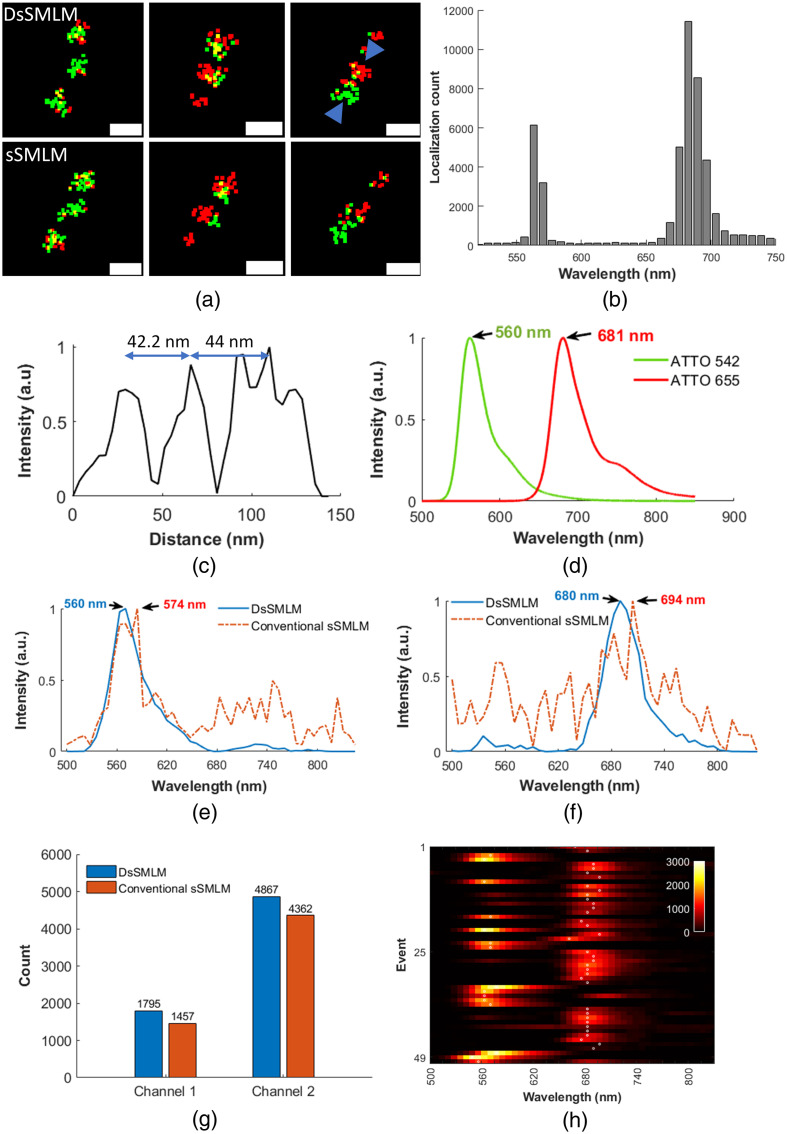
(a) DsSMLM and conventional sSMLM reconstructed simultaneous two-color super-resolution image of nanoruler sample labeled with ATTO 542 and ATTO 655 dyes. Scale bar = 100 nm. (b) The histogram of localization distribution with their corresponding spectral peak positions. (c) The line profile across the blue arrows in (a). The double arrow shows the distance between each cluster. (d) The ground truth normalized emission spectra of the labeled dyes. (e) and (f) Sample spectral profile from DsSMLM and conventional sSMLM reconstruction for the two dyes. (g) Comparison of predicted spectra peak count within the spectral window of 20 nm for DsSMLM and conventional sSMLM for channels 1 (ATTO 542) and 2 (ATTO 655). The mean number was obtained from 10K randomly selected spectra in 10 observations. (h) Fifty randomly selected DsSMLM reconstructed emission spectra with their spectral peak positions (white dots).

To further evaluate the broad applicability of the DsSMLM algorithm, we conducted reconstruction of DNA-point accumulation for nanoscale topography (DNA-PAINT)^59^ data for simultaneous multicolor imaging. DNA-PAINT is a recent SMLM technique that utilizes transiently binding, low-affinity fluorophore labels. The DsSMLM reconstructed images of a two-color nanoruler sample labeled with ATTO 542 and ATTO 655 dyes are shown in Fig. 9(a). Both DsSMLM and conventional sSMLM have excellent ability to distinguish two dyes labeled in three emitting points [three clusters in Fig. 9(a)]. Some emitters overlooked during conventional sSMLM reconstruction are detected well in DsSMLM reconstruction, as visualized in Fig. 9(a) (second and third columns), where some additional localizations are observed. These additional localizations likely originated from emitters with very low photon counts undetected by conventional sSMLM reconstruction. The yellow points are the overlapping localizations detected in each cluster. The localization counts histogram plotted with their spectral peak values in Fig. 9(b) show two grouped distributions representing two imaging channels. Localizations grouped around 561 nm are from the ATTO 542 dyes (channel 1) and around 680 nm are from the ATTO 655 dyes (channel 2). This indicates that a slightly higher number of localizations is detected from ATTO 655 dye. This is evident in Fig. 9(a), which has more red points than green ones. We used a 60 nm spectral window of 550 to 610 nm and 650 to 710 nm for ATTO 542 and ATTO 655 dyes to classify the localizations on the two imaging channels. The line profile across the blue arrows in Fig. 9(a) is shown in Fig. 9(d), which shows the mark-to-mark emitter distance of 42.2 and 44 nm, respectively. This verifies the known 40 nm distance between markers.

A sample emission spectra plot with spectral peak position from both imaging channels is shown in Figs. 9(e) and 9(f). The plots demonstrate that DsSMLM predicts accurate emission peaks closely matching the ground truth values of Fig. 9(d). The ground truth emission peak for ATTO 542 dye (channel 1) is 560 nm, and ATTO 655 (channel 2) is 681 nm. In both cases, DsSMLM effectively corrects the deviated peak value of around 14 nm.

In a separate analysis, we computed the spectral peaks for 10K randomly selected emitter samples for 10 random observations and observed a higher number of localization events on each imaging channel using DsSMLM compared to conventional sSMLM, as shown in Fig. 9(g). Specifically, DsSMLM identified an average of 1795 localizations with a s.d. of 34 on channel 1, while the conventional method detected an average of 1457 localization with a s.d. of 28. Similarly, on channel 2, DsSMLM distinguished notably higher localization with an average of 4867 localizations with a s.d. of 45, while the conventional method detected an average of 4362 localization with a s.d. of 31. The mean and s.d. counts were from a narrow spectral window of 20 nm for both imaging channels. Thus, using DsSMLM, an average of 3.38% and 5.05% higher localizations were revealed in channel 1 and channel 2, respectively. This improvement is due to the accurate prediction of spectral peaks using DsSMLM. This observation shows that the DsSMLM’s ability to predict precise spectral peaks improved the localization count in each channel. Also, such accurate prediction reduced the misrepresentation of localization to adjacent channels due to the deviation of the spectral peak, ultimately improving each channel’s image quality. It is worth noting that this kind of improvement plays a significant role in reducing the spectral cross-talk between imaging channels when performing simultaneous multicolor imaging using dyes with a narrow spectral separation. A few DsSMLM reconstructed spectra with their spectral peak positions (white dots) are shown in Fig. 9(h). The intensity of the spectra is augmented for better visualization. The emission curves in Figs. 9(e) and 9(f) are obtained using these spectra.

## 6 Discussion and Conclusions

In this work, we developed a DL approach called DsSMLM for the reconstruction of sSMLM imaging data. The sSMLM technique suffers from a limited photon budget due to the distribution of emission photons in the spatial and spectral image frame. However, our result demonstrated that with suitable training, DsSMLM can reconstruct high-quality super-resolution images for both label-free and fluorescence-labeled sSMLM imaging, even under challenging low photon budget conditions. DsSMLM utilizes two distinct CNN frameworks, namely U-Net and DCNN, which are trained on simulated data for two separate tasks: (1) spatial PSF localization and (2) spectral PSF enhancement. We first assessed the performance of two DL frameworks used in DsSMLM on simulated data. Then, we validated it through experimental label-free super-resolution imaging of ssDNA fiber data, achieving a spatial resolution of 6.22 nm. This represents a notable resolution enhancement of 2.28 nm compared to our previous finding using SICLON. The enhanced sub-10-nm resolution imaging efficiency afforded by DsSMLM enables the evaluation of unmodified DNA molecules, making it a valuable tool for imaging and studying chromatin by providing insights into native structures of DNA in cells without any exogenous labeling. We anticipate that DsSMLM will find extensive application in future studies of photophysics of DNA molecules and chromatin, enabling researchers to investigate their structure in its natural state.

Further, we demonstrated the applicability of DsSMLM for imaging and reconstructing the spatial organization of immunofluorescence-labeled histone markers (H3K9me3 and H3K4me3) at sub-diffraction resolution. Our finding also reveals that DsSMLM identifies up to 8.8% higher localization points than conventional sSMLM reconstruction. These additional localizations result in a high-density super-resolution image, adding more clarity to the structure being imaged. Moreover, the spectral analysis capability of DsSMLM enables us to reduce background noise and artifacts, facilitating a better understanding of the higher-order chromatin structures. To further delve into the exploration of nanoscale chromatin heterogeneity, we plan to extend DsSMLM imaging by integrating with the label-free partial wave spectroscopy^60^ using our newly developed multimodal imaging systems.^55^ This multimodal platform provides valuable insights into chromatin biology and enables the study of nanoscale chromatin structural changes.

Moreover, we demonstrate the simultaneous two-color imaging capability of DsSMLM using a synthetic DNA origami nanoruler sample. DsSMLM reconstruction revealed up to 5.05% higher localizations in two-color imaging than conventional sSMLM reconstruction. This confirms its enhanced discerning capability for more accurate detection of structures in two-color imaging. Our future work involves extending DsSMLM to enable simultaneous multilabel imaging of cellular structures to understand intercellular heterogeneity and chromatin biology. This includes simultaneous imaging of two or more histone markers. Accurate spectral signature identification offered by DsSMLM will be pivotal for such imaging, ensuring minimal spectral cross-talk and improving the quality of macromolecular structure visualization. In summary, we predict that DsSMLM capabilities will notably enhance simultaneous multilabel imaging techniques, enabling the study of complex chromatin structures with reduced artifacts.

While DsSMLM has promising capabilities, it is essential to acknowledge certain limitations in our approach. One such limitation is the imaging of densely packed histone protein. sSMLM usually requires very sparse and widely separated PSFs in spatial frames, ensuring that the corresponding spectra are not overlapped. Achieving such sparse PSFs in the image frames demands careful experimental design. Further, getting the sparse PSFs while imaging densely packed structures is extremely challenging. This is the major reason current sSMLM imaging in the literature is limited to simple cytoplasmic structures, such as tubulin, mitochondria, or peroxisome. Such limitations might be alleviated by developing algorithms for resolving the highly overlapping spectra, which will be investigated in our future studies. This is also extremely important in multilabel imaging because such overlapping spectra and smFSH introduce spectral cross-talk, causing cross-color contamination in the final image. Another existing challenge is identifying suitable brighter multiplexing dyes that can work in simultaneous imaging conditions. This challenge can be visualized in Fig. 9(b), where localization from ATTO 655 is significantly higher compared to ATTO 542 during simultaneous imaging conditions. We anticipate integrating DL techniques such as ours along with suitable brighter dyes with efficiently designed optical systems and experimental procedures will help to achieve high-quality multilabel super-resolution imaging of nanoscale cellular structures for understanding their interactions and biological implications.

## Data Availability

Code and data relevant to this article are not publicly available but may be obtained from the authors upon reasonable request.
